# Self-Supervised Anomaly Detection from Anomalous Training Data via Iterative Latent Token Masking

**DOI:** 10.1109/ICCVW60793.2023.00254

**Published:** 2023-12-25

**Authors:** Ashay Patel, Petru-Daniel Tudosiu, Walter H.L. Pinaya, Mark S. Graham, Olusola Adeleke, Gary Cook, Vicky Goh, Sebastien Ourselin, M. Jorge Cardoso

**Affiliations:** King’s College London

## Abstract

Anomaly detection and segmentation pose an important task across sectors ranging from medical imaging analysis to industry quality control. However, current unsupervised approaches require training data to not contain any anomalies, a requirement that can be especially challenging in many medical imaging scenarios. In this paper, we propose Iterative Latent Token Masking, a self-supervised framework derived from a robust statistics point of view, translating an iterative model fitting with M-estimators to the task of anomaly detection. In doing so, this allows the training of unsupervised methods on datasets heavily contaminated with anomalous images. Our method stems from prior work on using Transformers, combined with a Vector Quantized-Variational Autoencoder, for anomaly detection, a method with state-of-the-art performance when trained on normal (non-anomalous) data. More importantly, we utilise the token masking capabilities of Transformers to filter out suspected anomalous tokens from each sample’s sequence in the training set in an iterative self-supervised process, thus overcoming the difficulties of highly anomalous training data. Our work also highlights shortfalls in current state-of-the-art self-supervised, self-trained and unsupervised models when faced with small proportions of anomalous training data. We evaluate our method on whole-body PET data in addition to showing its wider application in more common computer vision tasks such as the industrial MVTec Dataset. Using varying levels of anomalous training data, our method showcases a superior performance over several state-of-the-art models, drawing attention to the potential of this approach.

## 1 Introduction

Anomaly detection aims to identify samples containing anomalous patterns that deviate from those seen in normal instances. The detection of these abnormal characteristics can be a challenging task in computer vision but is still crucial in a wide range of applications from medical imaging analysis [1, 19, 20] to manufacturing defect detection [3] and video surveillance [6]. One of the primary difficulties is the data requirements to train models. Usually, supervised anomaly detection requires large amounts of data with accurate annotations of the locations of said anomalies in the data. On the other hand, unsupervised methods require a sufficient number of instances that covers the normal variability of the data, but these instances must not contain anomalous patterns. Both these approaches thus require some form of labeling: segmentation maps for the supervised techniques and image-wide abnormality labels for the unsupervised approach, which can be challenging in specific applications. Lastly, for certain applications, all available images from the training set may present some form of local anomalies. A typical example of this is in medical imaging, where some modalities are collected only from patients with pre-existing suspicions of pathologies. Using certain modalities on healthy patients would be deemed unethical and potentially harmful due to radiation exposure. It is scenarios like this where obtaining a complete dataset without the presence of any anomalies for unsupervised training can be difficult, yet also greatly challenging and time-consuming to generate a large annotated dataset for supervised training given the size of 3D medical images.

A particular challenge for unsupervised anomaly detection is the development of training methods that allow the model to learn suitable features for the task without prior knowledge of the type and distribution of anomalies that may be present. Top-performing anomaly detection models commonly rely on models pre-trained on large datasets such as ImageNet to extract deep features [29, 21, 15] to circumvent the need for their model to learn relevant features. Even though effective, this generalised approach based on ImageNet falls short in specialised domains such as medical imaging [18]. Without pre-training, many methods rely on normative generative modelling, which can generate a pixel-wise anomaly score based on reconstruction residuals [5, 1]. However, the efficacy of such approaches is limited by the requirement of uncontaminated training data (i.e., normal only).

Until recently, the variational autoencoder (VAE) and its variants held the state-of-the-art for the unsupervised deep generative approach. For example, the spatial VAE approach [1] learns a normal data manifold by constraining the latent space to conform to a given distribution. This approach and varying autoencoder-only methods suffer from low-fidelity reconstructions caused by the latent-space information bottleneck and unwanted reconstructions on unseen anomalies. Recently, unsupervised anomaly detectors based on autoregressive transformers coupled with Vector-Quantized Variational Autoencoders (VQ-VAE) were proposed to overcome these issues [20, 19] showcasing state-of-the-art results in a range of 3D medical imaging anomaly detection tasks. In Pinaya et al. [20], the authors explore the advantage of tractably maximizing the likelihood of the normal data as well as the capacity of the attention mechanisms to better model the long-range dependencies of the training data. Patel et al. [19] takes this transformer-based method a step further by showing a greater resilience to training data containing anomalies through dropout and multiple sampling from the transformer used to generate a non-parametric Kernel Density Estimation (KDE) anomaly map. Even so, it was shown that the presence of anomalies in the training data was still detrimental to the model’s final performance when compared to anomaly-free training data.

Recently, self-supervised methods have shown promising results in computer vision tasks using data augmentation strategies on unlabelled data to mimic real anomalies. Cut-Paste [16] and Natural Synthetic Anomalies (NSA) [24] were shown to be effective for anomaly detection training paradigms. They generate synthetic anomalies by extracting random patches from the same or different training images and applying some form of transformation to the patch before blending it back into the images. Although impressive when trained on normal data alone, there is concern that the realism of such presented anomalies varies significantly from those seen in real data. The work in [17] attempted to tackle this problem via a self-trained knowledge distillation approach (STKD), in which a CutPaste model was trained on a mix of unlabelled normal and anomalous data to progressively filter out samples of high anomaly probability in the training data in an iterative manner. Although this method showcased improvements, it still displayed large performance degradation as the proportion of anomalous training increased and was only tested to low levels of anomaly contamination in the training data, levels that could likely be greatly exceeded in a medical imaging scenario.

For unsupervised generative models, given that the distribution of anomaly patterns is often unknown in advance, the primary goal is to train models to learn patterns of normal instances. Then during inference, anomalies are detected as deviations from the learnt representation of normality. Respectively for self-supervised anomaly detection, the assumption is that all anomalies present within the training data are synthetic and clear deviations from the normal data, and as such, the model learns to locate these synthetic anomalies. In both approaches, training data is assumed to be normal. However, there is little evidence and research to showcase the efficacy of said approaches when normal training data alone is unattainable. The difficulty is task-specific. For example, it may be infeasible to review long videos in video surveillance data to ensure each frame is without anomalous objects. For medical imaging, this can be an ethical challenge in that certain imaging modalities, i.e. those resulting in radiation exposure, should not be used on patients without prior suspicions of pathologies. In such scenarios, you may find datasets with a heavy bias towards containing anomalous samples with little to no normal training samples [19].

Given the prevalence of datasets contaminated with outliers and anomalies, robust statistical approaches have been implemented, however, there are limited applications of such approaches to imaging data. Some of the earliest work tackled simple regression problems with outliers present. Notable solutions include Least Trimmed Squared [23], an iterative fitting approach that aims to minimise the sum of squared residuals to a subset of the data and Least-Median-of-Squares [22], a fitting technique that seeks to minimise the median of squared residuals. Although effective for model fitting in the presence of anomalies, these approaches are unfeasible and unsuitable for higher dimensional imaging problems. One perhaps more suited robust approach is Random Sample Consensus (RANSAC) [12]. This method fits a model to random subsets of the data and returns the model with the best fit (or least error) to its specific subset. The reasoning behind this is the assumption that a model will be able to fit well to inliers with minimal error whilst producing a higher error when fit to data with outliers. Similar to work like STKD however this approach is only suitable for datasets with lower levels of outliers and not high levels of anomalous samples, a specific challenge we are attempting to tackle in medical imaging where all the data could potentially contain anomalies. However the concept of iterative/multiple training cycles and robust fitting techniques to data with outliers is of importance and where we take inspiration for our approach.

**Figure 2:**
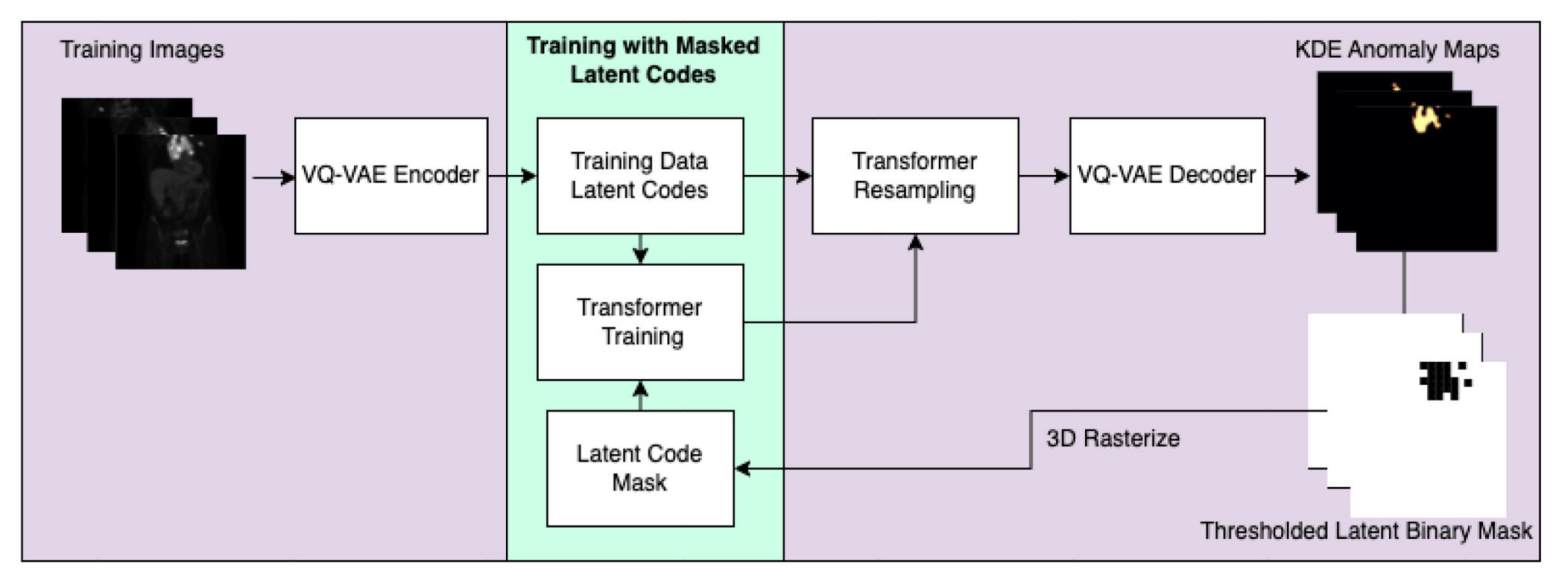
Proposed Iterative Latent Token Masking training pipeline. Training samples are quantized using the trained VQ-VAE’s encoder, where a trained Transformer replaces anomalous tokens with multiple samplings. After decoding multiple latent codes with dropout, a KDE anomaly map is generated. This map is then thresholded and downsampled to give a binary mask of the same dimension as the latent space. These binary masks are then rasterized and used to mask tokens in the next Transformer training (as shown in the green box).

Given such challenges, we propose Iterative Latent Token Masking (ILTM), a robust statistical approach to self-supervised anomaly detection that uses the VQ-VAE - Transformers pipeline with self-supervised token masking (Figure 2). On 3D whole-body PET anomaly detection, we showcase that our method outperforms leading methods and presents minimal deterioration in performance when faced with highly anomalous training data. Furthermore, to show-case the efficacy of this approach on wider computer vision tasks, we also report the results of the proposed on the 2D industrial MVTec dataset.

## 2 Background

The driving components behind the proposed method use VQ-VAE and Transformer models to learn the probability density function of the training images. To use Transformer models, images must be presented in a 1D sequence of values that are ideally categorical. Using voxel values alone flattened into a sequence is infeasible, given the size of the images. Based on previous work [19, 20, 26], we use a VQ-VAE model to learn a discrete latent representation of the images that are then fed into the Transformers.

### 2.1 VQ-VAE

The VQ-VAE offers state-of-the-art reconstruction results while providing an ideal encoding of images in a discrete format for the Transformer network. The VQ-VAE is composed of an encoder that maps an image *x* ∈ ℝ^*H*×*W*×*D*^ onto a smaller latent representation $Z\in {\mathbb{R}}^{h\times w\times d\times n_{z}}$ where *n*_*z*_ is the latent embedding vector dimension. This representation *Z* is then passed through a quantization block where an element-wise quantization is done to map each feature column vector to its nearest codebook vector. Each spatial code $Z_{ijl}\in {\mathbb{R}}^{n_{z}}$ is then replaced by its nearest code-book element $e_{k}\in {\mathbb{R}}^{n_{z}},k\in 1,\ldots ,K$ where *K* denotes the codebook vocabulary size, thus obtaining *Z*_*q*_. Given *Z*_*q*_, the VQ-VAE decoder then reconstructs the observations $\hat{x}\in {\mathbb{R}}^{H\times W\times D}$. For detailed formulations of losses, architectures, and training processes, please refer to Appendix A.

### 2.2 Transformer

After training a VQ-VAE model, the next stage is to learn the probability density function of the discrete latent representations from the training data. Using the VQ-VAE encoder and quantization block, we can obtain a discrete representation of the latent space by replacing the codebook elements in *Z*_*q*_ with their respective indices in the codebook yielding *Z*_*iq*_. To model the imaging data, we require the discretized latent space *Z*_*iq*_ to take the form of a 1D sequence *s*. In this work, we used a raster scan ordering to transform *Z*_*iq*_ into *s*. The Transformer is then trained to maximize the log-likelihoods of the latent sequence tokens in an autoregressive manner. By doing this, the Transformer can learn the codebook distribution for any position *i* within *s* with respect to previous codes *p*(*s*_*i*_) = *p*(*s*_*i*_|*s*_<*i*_). For Transformer formulations in addition to architectural and training details, please refer to Appendix B.

By learning the conditional probability of tokens in the sequence at a given location with respect to all previous tokens in the sequence (*p*(*s*_*i*_) = *p*(*s*_*i*_|*s*_<*i*_)), the Transformer can single out low probability or anomalous tokens during inference, a key mechanism for the anomaly detection and token masking pipeline used in this work.

## 3 Method

### 3.1 Anomaly Detection with M-estimators

A robust statistics perspective is adopted to account for anomalies in the training data. An M-estimator, a class of robust estimator, can be used to estimate the parameters *θ* of a model that maximize the log-likelihood function of the data. Given the objective function for training a transformer, the transformer takes the form of our M-estimator in this scenario. The objective is to learn the distribution of the latent tokens *p*(*s*_*i*_) = *p*(*s*_*i*_|*s*_<*i*_) by maximizing the log-likelihood of the training data expressed as:

(1) $$ℒ(\theta )=\sum_{i=1}^{N}logp\left(Z_{i},\theta \right)$$

where N represents the number of training examples, *Z*_*i*_ is the quantized latent code for the *i*^*th*^ sample, and *θ* represents the parameters of the transformer model.

Although traditionally the M-estimator would seek to minimize the influence of outliers or anomalies in the data, this cannot be guaranteed in this setting. However, from prior research [19] and knowledge of the spatial distribution of anomalies, we can assume that the influence of outliers will be such that the estimator will still adequately model the inlier distribution. To improve on this however we can use a second objective function to identify and remove outlier tokens, to improve fitting in the presence of anomalies.

Letting $f\left(x_{z_{j}}^{i},\theta \right)$ denote the anomaly score for the *j*^*th*^ latent code in the *i*^*th*^ image and its corresponding quantized latent code. This function defines the likelihood or anomaly level of that token. To do so. we need a robust method for determining outliers in the training data.

This is inspired by the Robust Kernel Density Estimation (RKDE) [14] approach of combining an M-estimator approach with Kernel Density Estimation. The work in [14] states that as the sample mean is sensitive to outliers, we estimate the KDE robustly via M-estimation. Inspired by this work, we employ M-estimation through our trained transformer to generate the data to calculate our KDE to calculate the outlierness of latent tokens. As previously stated, the spatial distribution of anomalies would reduce the influence of outliers in the training of the Transformer model, and therefore an M-estimator that is resilient to outliers, to a certain degree. As such, we believe we can reduce the influence of outlier tokens by resampling new tokens from the transformer when faced with low likelihood tokens.

To do so, we generate multiple reconstructions of input images, with low likelihood tokens resampled from the Transformer.

This is done through the inferred likelihoods that represent the probability of each token appearing at a certain position in the sequence with respect to all previous tokens in the sequence *p*(*s*_*i*_) = *p*(*s*_*i*_|*s*_<*i*_). This can then be used to single out tokens with low probability, i.e. tokens that might be anomalous. We can then highlight those with likelihoods below a given arbitrary threshold to generate a binary resampling mask which indicates anomalous tokens *p*(*s*_*i*_) < *t* where *t* is the resampling threshold. In this work, a value of 0.005 is used for *t* based off [19]. Using this resampling mask, anomalous tokens can be replaced with higher likelihood tokens by resampling from the Transformer. In doing so, these high-likelihood tokens are more likely to represent non-anomalous data in the reconstructed image. We can then reshape the “healed” sequence back into its 2D or 3D quantized representation to feed back into the VQ-VAE to generate a non-anomalous reconstruction *x*_*r*_ without anomalies. However, instead of generating one reconstruction, we sample multiple times for each position *i* with a likelihood *p*(*s*_*i*_) < *t*, i.e. below the resampling threshold. In each of these samplings, the Transformer outputs the likelihood for every possible token at position *i*. Based on these probabilities we can create a multinomial distribution showcasing the probability of each token appearing at position *i* in the sequence from which we can sample from. When this is applied over all low-likelihood tokens, this gives multiple “healed” latent spaces. Each of these latent values is then fed into the VQ-VAE decoder, where each is then decoded multiple times with dropout. In doing so, we get many possible normal realizations of the original image. At this point, a KDE is fit independently at each voxel position in the image to generate an estimate of the probability density function *f*. Letting (*x*_1_, …, *x*_*n*_) be the intensity values at a given voxel position across reconstructions, we can generate an estimation for the shape of the density function *f* for pixel *x* as:

(2) $${\hat{f}}_{h}(x)=\frac{1}{nh}\sum_{i=1}^{n}K\left(\frac{x-x_{i}}{h}\right)$$

where *K* is a given kernel shape and *h* is a smoothing bandwidth calculated via the Silverman method [25] as:

(3) $$h={\left(\frac{4{\hat{\sigma }}^{5}}{3n}\right)}^{1/5}+\epsilon$$

where $\hat{\sigma }$ represents the standard deviation across reconstructions at the given pixel position and *ϵ* is a scalar regularisation parameter chosen as 12.75 for the MVTec dataset and 0.05 for AutoPET based off [19]. From this, we can then score each voxel position from its estimated density function at the intensity of the original image at the voxel level to generate the KDE anomaly map.

For a given voxel position (*i, j, k*), the KDE-based log-likelihood score (*L*) is calculated as:

(4) $$L_{ijk}=log\left[\sum^{\;}\frac{K\left(x_{n}(i,j,k),x(i,j,k)\right)}{h}\right]$$

where K is the kernel function, *x*_*n*_ (*i, j, k*) represents the pixel intensity of reconstruction *n* at position (*i, j, k*), *x*(*i, j, k*) represents the original pixel intensity at position (*i, j, k*), and h is the bandwidth parameter. Voxels with log-likelihood scores below a specified threshold are classified as outliers. For this work, we used a likelihood of 0.005, however this value was arbitrarily chosen and has not been subject to any tuning on separate labelled datasets making the approach fully unsupervised and self-trained. Further tuning of this value could generate improved results.

The implementation of the KDE approach in this work used sampling 60 times for each anomalous token in *s*. Then, each of the 60 healed latent representations is decoded with dropout five times to yield 300 reconstructions used to calculate the density functions.

### 3.2 Iterative Latent Token Masking

Our proposed self-supervised anomaly masking method consists of an iterative method of training our M-estimator - the transformer, and using the KDE anomaly detection method on the training data to create intermediate anomaly maps to guide the token masking (Figure 2). The method uses the fact that within the training data we can assume that the inliers belong to a given distribution independent and separate from the outliers. We thus want to be able to train the transformer such that *θ*_*in*+*out*_ approximates to *θ*_*in*_ with minimal discrepancy, where *θ* represents the transformer model parameters trained on inliers + outliers and only inliers respectively. Of course this error is still present and we require a way to move *θ*_*in*+*out*_ towards *θ*_*in*_. For this, we use the robust anomaly detection described in section 3.1 to highlight suspected anomalies in the training data and then retrain the model while removing their influence during training. In doing so and repeating this process over *m* iterations, we expect:

(5) $$\underset{m\to \infty }{\text{lim}}\theta_{in+out{\;}_{m}}=\theta_{in\;}$$

Of course, retraining a large number of times is both inefficient and unnecessary, and we can stop the iterative process once a suitable convergence has been displayed. Like self-training methods [11, 17], we use an initial training iteration to generate pseudo labels for the training data. However, unlike this work in [11] that uses a small subset of labelled date we use the entire anomalous training dataset in a fully unsupervised way with no labels. Additionally, these pseudo labels are used to mask or remove the influence of suspected anomalies in the training data so that we can emulate a fully unsupervised learning approach with normal data only – this is opposed to learning from the pseudo labels directly. We can repeat this process several times, where the model can perform better over each iteration, highlighting and masking anomalies in the training data to a point where the data is almost entirely void of anomalies. In contrast to the STKD method in [17] that also uses no labels, instead of removing anomalous samples entirely from the training set, we simply mask anomalies and learn from the normal areas in the image, therefore retaining our training data size and making our approach more robust to higher levels of anomalies - an observed difficulty presented in the STKD approach.

The core concept behind our approach is token masking in the Transformer. This concept is not unfamiliar in that the nature of autoregressive self-attention requires token masking during training to prevent look-ahead bias of future values in the sequence [28]. Similarly, masking has been a common training mechanism in language and generative modeling for natural images [9, 4]. The approach from our study more closely resembles the masking used in autoregressive self-attention as we are trying to remove the influence of the masked tokens during training.

To do this, we perform anomaly detection on the training data to generate a KDE map for each training sample and therefore segmentations of the anomalies in this training cycle. Using an optimal Transformer resampling threshold based on [20, 19] and an arbitrary KDE likelihood threshold of 0.005, we can turn our KDE map into a binary segmentation map. This threshold is an arbitrary choice that has undergone no parameter tuning. Further exploration of this value is beyond the scope of this work. In doing so, we can generate a binary segmentation map for each sample in the training data without using any labelled data. We can take away from the work in [20, 19] that the spatial information in the image space directly correlates to the information held in the latent space. As illustrated in Figure 2, we can downsample each segmentation map to the latent space dimension, giving us a binary mask over the tokenized space for the specific training image. This can then be passed to the Transformer to ignore said token during the next round of training in addition to the loss calculations.

Given the tokenized sequence *s* and list of anomalous tokens to be masked *s*_*masked*_ the Transformer can now autoregressively learn the likelihood for a given code at a given position *i* with respect to all previous tokens minus those that have been masked, i.e. $p\left(s_{i}\right)=p\left(s_{i}|\left(s_{<i}\setminus s_{masked{\;}_{<i}}\right)\right)$ Our training objective for the transformer from 1 trained on the latent representations *Z* with masks *M* now becomes:

(6) $$ℒ\left(\theta^{*}\right)=\sum_{i=1}^{N}logp\left(Z_{i}\setminus M_{i},\theta^{*}\right)$$

During training, token masking works by assigning a negative infinity value to masked tokens due to the softmax step of the attention mechanism. Given our input sequence *s* of length *l*, the dot product of the Key and Query value gives us a square matrix of shape *l* × *l*. For each row or column corresponding to the position of a masked token, that entire row and column will take a value of negative infinity, which after the softmax function will give a value of 0. This removes the influence of these tokens in the attention mechanism. Similarly, the cross-entropy loss function for the Transformer is also adapted to ignore the influence of the masked tokens. Through these actions, we remove the influence of these suspected anomalous tokens in predicting others, in addition to removing their influence on the learnt probability distributions. Once the Transformer is trained with masking, the process is repeated with the newly trained. We repeat this process until we see convergence in the number of tokens masked in the training data.

This approach relies on an adequate anomaly detection performance in the first iteration. Given highly anomalous training data however, a higher resampling threshold may be required to achieve said performance given the prevalence of anomalies in the training data increasing their likelihood during inference. This requirement, however, would be reduced over iterations as our method would mask anomalies during training. Therefore, we propose a second method in which the same resampling threshold determined on the first iteration is reduced by a given percentage over each iteration. For this work, we arbitrarily chose a reduction of 12.5% for each iteration from an initial threshold of 0.005. This threshold was chosen using parameters seen in prior research [20, 19] and has not been subject to further tuning, albeit we could see further improvements should a more detailed study on this chosen threshold be conducted.

### 3.3 Data

#### AutoPET - 3D Whole-Body PET Dataset

Our primary medical imaging dataset to showcase the ability of our model in a scenario where non-anomalous training data is often difficult to obtain makes use of autoPET - a 3D whole-body Positron Emission Tomography (PET) dataset [13, 8]. This dataset consists of 1014 PET scans with 430 healthy scans, with the remaining containing some form of lung cancer, lymphoma, or melanoma. We create a validation and testing dataset of 20 and 50 subjects, respectively. The remaining data is then used to create the training data where training datasets of 425 subjects with anomalous to healthy subject ratios ranging from 0% to 100% (a common scenario in oncology imaging).

#### MVTec Dataset

For our second experimental dataset to showcase the use of this work in a wider computer vision setting, we used the MVTec dataset, a dataset widely adopted to evaluate anomaly detection methods in industrial imaging settings [3]. As we aim to showcase the efficacy of unsupervised learning approaches when confronted with anomalous training data, we predominantly used the test data set to generate our training, validation, and test sets. The validation and testing datasets have 5 and 20 samples per category respectively. We then used the remaining anomalous samples to create our training data. Combined with the remaining normal samples, through a combination of under and over-sampling a range of datasets from 0% to 80% anomalous were created. Given the limited anomalous data in the toothbrush and transistor categories, these were excluded from our study. All images were resized to 256×256 pixels, and the same augmentations as used in the AutoPET dataset were used minus elastic deformations.

## 4 Results and Discussion

We conduct our experiments on the hold-out testing set of 20 samples per category of the MVTec dataset and 50 samples for the PET data. For evaluation metrics, we report the pixel/voxel-wise area under the per-region overlap curve (AUPRO) and the pixel/voxel-wise area under the receiver operating characteristic curve (AUROC) in addition to the best achievable DICE score, obtained using a greedy search for thresholding the residual/density score map. Anomaly detection is run using the same method and transformer resampling threshold as described in section 3.1.

### 4.1 Experiment 1: Anomaly Detection over Training Iterations

**Figure 3:**
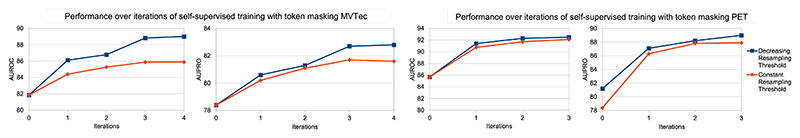
Anomaly Detection Performance of our proposed method over training cycles for the MVTec data (left) and PET data (right). Results showcase performance using a constant Transformer latent token resampling threshold (red) and a decreasing threshold over iterations (blue).

In the first experiment, we aim to showcase the improvement in model performance over iterations of the proposed training scheme. We also investigate the Transformer resampling threshold used in the anomaly detection method. As described in Section 3.2, we run two models, one with a fixed and one with a decreasing resampling threshold. This is run on the training dataset with 80% and 100% anomalous data for MVTec and PET, respectively. The results over the training iterations are in Figure 3. The stopping criterion was based on the number of tokens categorised as anomalous at each iteration. The stopping criteria was hit when this number was within 1% of the previous iteration. Testing at each iteration was not performed until this stopping criterion was met and all training had finished.

These results show the impact of reducing the threshold, although more evident in the MVTec data. This indicates the trade-off between the detection rate and the false positive rate. During initial iterations, a higher resampling threshold is preferred to detect anomalies and remove their influence in future iterations. As iterations are completed, the influence of said anomalies is masked, and the requirement for a higher resampling threshold is reduced. By reducing this threshold, the model can reduce false positives and masking of non-anomalous tokens, allowing the Transformer to train better with more refined masking. Given this improvement, this method is used in Experiment 2.

### 4.2 Experiment 2: Anomaly Detection with Varying Anomalous Training Data

The second experiment explores the effect of the proportion of training data containing anomalies on model performance. We use cases up to 80% anomalous in MVTec to rigorously stress test our method. Additionally, we use cases up to 100% anomalous for AutoPET given the high possibility of these levels in a medical imaging environment. To showcase the efficacy of our approach, we select several state-of-the-art models to compare results with. This includes a Dense AE and Dense VAE [2, 1] from an unsupervised reconstruction-only approach in addition to CutPaste [16] and NSA [24] from self-supervised work. We also compare our work to that of STKD a self-trained method that showed state-of-the-art performance when trained on anomalous training data [17]. Additionally, we compare our approach to that of the same model without ILTM for training, as proposed in [19], to highlight the added contribution of the token masking methodology.

**Figure 4:**
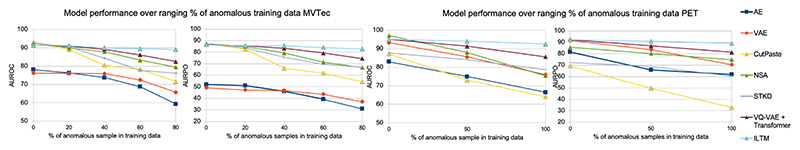
Anomaly Detection Performance of our proposed methods along with state-of-the-art models for comparison showing change in AUROC and AUPRO with varying levels of anomalous training data.

**Figure 5:**
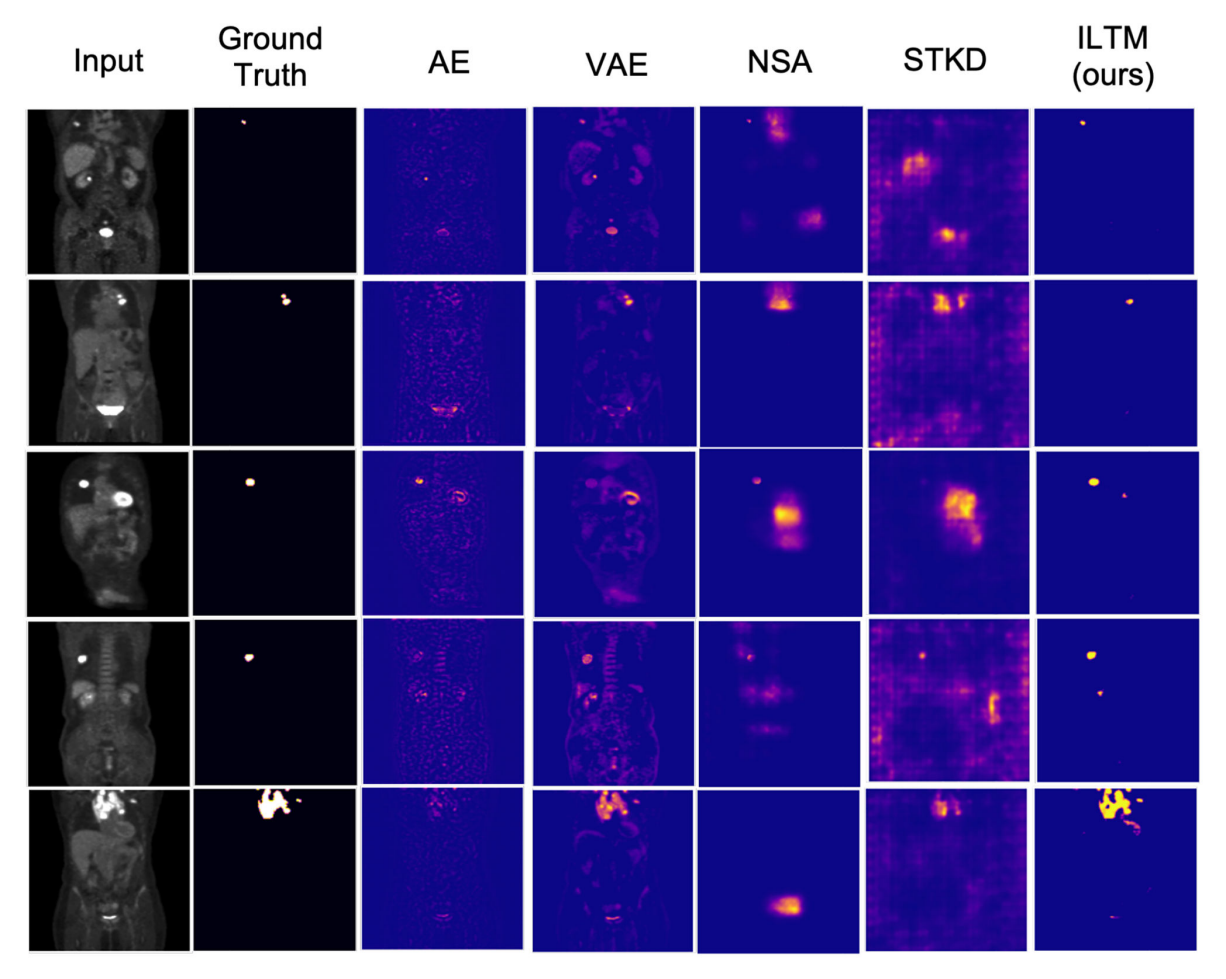
Rows from top to bottom display (1st) input image; (2nd) ground truth segmentation; (3rd) anomaly map as the residual for the AE, (4th) VAE; (5th) abnormality map output for NSA, (6th) STKD; (7th) abnormality map as a KDE using our self-supervised training approach. Results are provided for 5 random samples from the PET dataset

Figure 4 shows a consistent story across the baseline models. From the unsupervised reconstruction-based models, we see significant reductions in performance as anomalous training increases. This is brought about by the model’s ability to reconstruct anomalies seen during training, visualised in both Figure 6 and Figure 5. For NSA and Cut-Paste, the story is more telling. The initial performance of NSA at 0% anomalous training performs better than our method on both the PET and industrial data. However, as the anomalous training percentage increases, their performance significantly reduces, falling below our approach’s performance at only 20% contamination. This is likely because the model learns to detect synthetic anomalies while treating real ones as normal. As such, we see a reduction in performance, such that our method outperforms NSA by 9.5 AUROC and 16 AUPRO on MVTec and 17 AUROC and 14.2 on PET data for the highest contamination levels. Similarly with STKD, although an improvement on CutPaste, performance still greatly deteriorates as much as 9 AUROC from just 0-40% anomalous data in comparison to a reduction of 1.5 AUROC in our proposed method. For STKD this becomes worse as the levels of anomalies increase. This is likely due to the method removing anomalous samples limiting the training data available - this is opposed to our method, which does not remove anomalous samples but instead removes the influence of anomalous areas in a sample and still learns from the normal areas. Our approach using iterative training with token masking showcases a superior performance as the amount of anomalous training data increases. With anomaly contamination as low as 20%, our approach shows the best performance, with the gap only increasing as the anomalous ratio increases. We can see from Figure 4 that at the highest levels of contamination, there is little reduction in performance with less than a 2.5 and 3.7 reductions in AUROC and AUPRO for both datasets. Qualitatively Figure 5 shows limited issues with detection.

Furthermore, a breakdown of each model’s performance with training data that is 80% anomalous for MVTec and 100% anomalous for PET can be seen in Table 1. A full breakdown by category and further qualitative results for the MVTec dataset can be seen in C.

## 5 Conclusion

In this study, we propose a training mechanism for anomaly detection with training data heavily contaminated with anomalous samples. Using a Transformer-based anomaly detection approach with KDE anomaly maps, we utilise the token masking capabilities of Transformers to mask out anomalous data during training iterations, generating a method whose performance is highly unaffected by the presence of anomalous training data. The proposed methods showcase significantly improved performances over current state-of-the-art models in self-trained, self-supervised and fully unsupervised approaches, some of which even showcase greater performance when trained on normal data alone. We hope that this work can be used to alleviate data restrictions on training data. This can be important in fields like medical imaging where data can be prone to have many anomalies, or even to alleviate the need to check and screen training data to ensure only normal data is used. In doing so, we can increase the potential size of data at our disposal when used for training without added data processing time. We hope this work will inspire further investigation into anomaly detection without normal data, further expanding on this rarely explored field.

## Supplementary Material

Supplementary Appendix

## Figures and Tables

**Figure 1:**
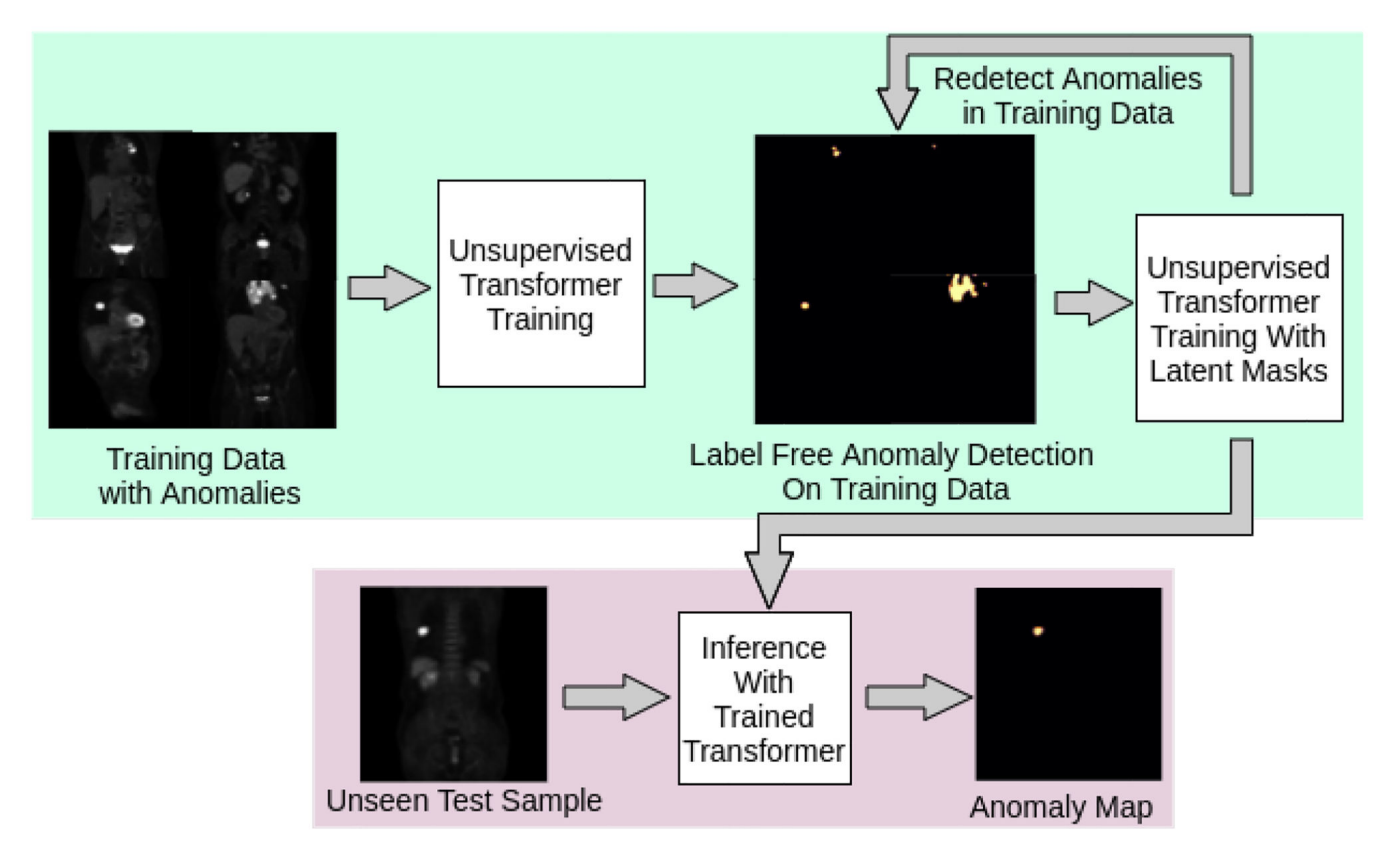
Our approach can take anomalous training data without prior segmentation labels and train a Transformer to progressively remove the influence of anomalies from training such that, during inference, the model can detect new anomalies.

**Table 1:** Anomaly detection results of the proposed method in comparison to baseline comparisons. For each dataset, AUROC (top row), AUPRO (middle row) and DICE (bottom row) are given along with the respective standard deviation. Best-performing methods are highlighted in boldface.

	AE [1]	VAE [1]	CutPaste [16]	NSA [24]	STKD [17]	VQ-VAE + Transformer [19]	ILTM
MVTec	59.3	65.4	72.2	79.6	76.1	82.7	**89.1**
	30.9	37.3	54.8	66.8	67.1	74.3	**82.8**
	24.3	26.4	32.7	38.5	39.0	58.7	**65.2**
AutoPET	66.5	76.1	63.9	75.6	78.7	85.7	**92.6**
	63.7	70.5	32.8	74.8	76.4	82.1	**89.0**
	41.1	43.5	38.4	44.8	46.2	59.6	**68.3**
