# Supplementary material for: Self-Supervised Anomaly Detection from Anomalous Training Data via Iterative Latent Token Masking
Source: IEEE Int Conf Comput Vis Workshops. Author manuscript; Available in PMC 2024 Aug 28. (PMC7616405; doi:10.1109/ICCVW60793.2023.00254)
Supplement: Supplementary Appendix [file EMS197106-supplement-Supplementary_Appendix.pdf]

A. VQ-VAE Implementation

The VQ-VAE model trained used the same model parameters across each category on the MVTec Dataset. Several different codebook sizes were experimented with, with the codebook that resulted in the lowest average reconstruction error as the final codebook to be used (note this may not be optimal for anomaly detection performance). The VQ-VAE model was trained using a jukebox loss that is given as:

$$L_{VQVAE} = \|(\mathbf{x} - \hat{\mathbf{x}})\|_2^2 + \||STFT(\mathbf{x})| - |STFT(\hat{\mathbf{x}})|\|_2^2 + \beta \|z_e(\mathbf{x}) - sg[\mathbf{e}]\|_2^2 + \|sg[z_e(\mathbf{x})] - \mathbf{e}\|_2^2 \quad (7)$$

where sg stands for a stop gradient operator to stop gradients from flowing back into their argument. The loss used from [10] uses a spectral loss component that is based on the magnitude of the Fourier Transformer of the original and reconstruction image. From equation 7 the first term seen is the L2 pixel loss, whilst the second term represents the spectral loss between the original and reconstruction. Here SFTF stands for the short-time Fourier transform. The third term is the commitment cost to ensure the encoder commits to the codebook. The final term is to move the codebook embedding vectors towards the output from the encoder. In the implementation we replace this final term with an exponential moving average update for the codebook as implemented in [27]. During training, a β of 0.25 was used.

The architecture used for the VQ-VAE model for the MVTec Dataset used an encoder consisting of three downsampling layers that contain a convolution with stride 2 and kernel size 4 followed by a ReLU activation and 3 residual blocks. Each residual block consists of a kernel of size 3, followed by a RelU activation, a convolution of kernel size 1 and another ReLU activation. Similar to the encoder, the decoder has 3 layers of 3 residual blocks, each followed by a transposed convolutional layer with stride 2 and kernel size 4. Finally, before the last transposed convolutional layer, a Dropout layer with a probability of 0.05 is added. The VQ-VAE codebook with optimal reconstruction was found to have 256 atomic elements (vocabulary size), each of length 32. To train the VQ-VAE network, we used an ADAM optimiser with a learning rate of 1e-4 and an exponential learning rate decay with a gamma of 0.9999. Training was run for 20,000 epochs with a batch size of 16. During training, the data was augmented with Gaussian noise, contrast adjustment, intensity shifts, translations, rotations and scaling. The VQ-VAE model used for the 3D PET data consisted of the same architecture with 3D kernels. Additionally the codebook has a 256 vocabulary size each of length 128. This model was trained for 1000 epochs with a batch size of 3. In addition to the augmentations used for the MVTec dataset, elastic deformations were also carried out during training.

B. Transformer Implementation

Once the VQ-VAE model was trained, and training data could be encoded, a Transformer could then be trained on the encoded images, using their discrete latent representations. The self-attention mechanism is best described as a mapping of intermediate representations of three position-wise linear layers onto three representations denoted by the Value (V), Key (K) and Query (Q) [28]. With d_k denoting the dimension of the key, query and value vectors, the attention mechanism is calculated as follows:

$$Attn(Q, K, V) = softmax\left(\frac{QK^T}{\sqrt{d_k}}\right)V \quad (8)$$

The Transformer success relies on the self-attention mechanisms employed to capture the interactions between inputs in the sequence regardless of their relative position to one another. This relies on the inner product between elements of the sequence and as such the network scales quadratically with sequence length. This is a key limitation when applied to image data. In this work, we use the Performer variant that proposed a linear generalized attention offering a more scalable approach [7]. The performer makes use of multi-headed self-attention. This aspect in the network is several attention layers run in parallel with their outputs concatenated and fed through a linear layer.

The performer used in this work for the MVTec dataset corresponds to a decoder Transformer architecture with 16 layers, each with 8 heads, and an embedding size of 32. For PET data, the performer had 14 layers, each with 8 heads and an embedding size of 256. To train the network, we used an ADAM optimiser with a learning rate of 1e-3, an exponential learning rate decay with a gamma of 0.9999 and cross-entry loss. Furthermore the embedding, feed-forward and attention mechanisms within the network all had a dropout probability of 0.1.

Given the nature of the recursive training, and required inference on training to generate the latent code masks, to avoid extra overfitting to the original training data resulting in poor anomaly detection performance on the training data, the data fed in to the Transformer during training was augmented data, using the same data augmentations used in VQ-VAE training, i.e. Gaussian noise, contrast adjustment, intensity shifts, vertical and horizontal translations, rotations, scaling (and elastic deformations in the case of the PET data). The training was then performed over 80 epochs with a batch size of 1.

C. MVTec Anomaly Detection Performance

Table 2. Anomaly detection results of the proposed method in comparison to state-of-the-art comparisons. For each dataset category, AUROC (top row) and AUPRO (bottom row) are given along with the respective standard deviation. The best-performing method is highlighted in boldface.

Category	AE [1]	VAE [1]	CutPaste [16]	NSA [24]	VQ-VAE + Transformer [19]	ILTM
bottle	45.9 ± 11.5	65.5 ± 12.2	82.0 ± 6.3	91.1 ± 3.9	87.2 ± 5.3	**95.1** ± **1.4**
	2.8 ± 19.6	39.3 ± 14.1	65.7 ± 12.2	81.0 ± 6.7	81.9 ± 6.7	**91.0** ± **3.4**
pill	79.6 ± 10.0	83.1 ± 8.9	74.8 ± 8.2	87.0 ± 5.3	84.5 ± 4.8	**92.6** ± **2.8**
	49.0 ± 19.2	55.4 ± 19.4	69.5 ± 11.1	79.3 ± 8.6	78.5 ± 9.6	**88.5** ± **6.9**
screw	54.0 ± 17.0	75.2 ± 17.5	80.8 ± 6.2	81.2 ± 7.5	81.2 ± 8.0	**89.9** ± **4.9**
	22.2 ± 22.9	36.0 ± 21.4	73.1 ± 11.0	76.1 ± 11.8	71.4 ± 13.1	**82.7** ± **8.7**
cable	46.0 ± 13.1	59.3 ± 15.4	69.4 ± 7.4	84.3 ± 6.5	85.2 ± 6.7	**87.9** ± **6.1**
	15.2 ± 13.6	23.9 ± 13.6	34.6 ± 13.6	68.5 ± 13.2	73.9 ± 13.2	**78.3** ± **16.4**
hazelnut	82.1 ± 12.8	68.7 ± 12.0	78.9 ± 9.5	82.5 ± 6.8	89.5 ± 10.6	**97.5** ± **1.1**
	72.1 ± 12.7	45.5 ± 19.8	58.6 ± 12.7	63.7 ± 10.6	82.1 ± 6.1	**93.1** ± **3.6**
zipper	62.3 ± 9.5	66.9 ± 8.3	86.1 ± 8.1	82.9 ± 8.0	80.4 ± 7.6	**89.1** ± **5.1**
	41.1 ± 12.4	52.4 ± 10.2	77.5 ± 11.8	74.1 ± 9.3	73.6 ± 14.9	**81.1** ± **6.2**
metal nut	78.3 ± 6.2	77.2 ± 8.1	75.1 ± 9.1	84.1 ± 4.7	82.1 ± 6.1	**90.1** ± **3.4**
	61.2 ± 12.4	59.1 ± 14.1	59.1 ± 15.1	70.1 ± 9.1	75.1 ± 9.8	**83.5** ± **51.2**
tile	53.6 ± 9.2	46.0 ± 14.5	68.6 ± 13.8	**79.2** ± **9.3**	72.1 ± 12.0	72.2 ± 14.1
	21.3 ± 15.8	15.2 ± 18.7	48.5 ± 18.4	**62.5** ± **15.2**	56.9 ± 18.2	57.9 ± 21.4
wood	49.5 ± 15.7	59.2 ± 7.6	69.0 ± 7.0	74.7 ± 7.7	81.7 ± 6.1	**88.2** ± **4.1**
	18.9 ± 22.4	34.1 ± 7.5	46.3 ± 17.5	55.1 ± 9.7	75.7 ± 7.0	**90.2** ± **4.9**
leather	51.8 ± 9.0	72.5 ± 20.5	53.3 ± 15.2	64.0 ± 7.5	90.8 ± 3.0	**97.1** ± **1.6**
	25.4 ± 13.0	48.5 ± 25.2	35.3 ± 21.6	51.4 ± 14.4	83.7 ± 6.4	**93.8** ± **1.9**
carpet	52.5 ± 7.8	51.3 ± 3.3	54.6 ± 14.2	61.4 ± 14.9	76.8 ± 15.0	**80.4** ± **8.5**
	18.6 ± 9.8	16.7 ± 3.9	24.7 ± 19.0	45.1 ± 19.4	65.8 ± 21.5	**71.3** ± **14.7**
grid	55.9 ± 10.7	59.4 ± 8.6	73.6 ± 11.7	82.5 ± 7.1	81.5 ± 12.1	**89.0** ± **4.1**
	22.8 ± 15.1	20.9 ± 8.9	65.3 ± 14.8	75.2 ± 8.6	72.5 ± 6.2	**82.0** ± **4.7**

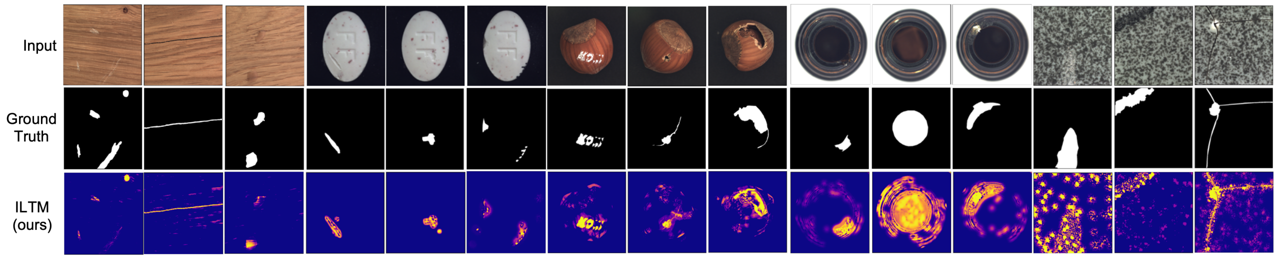

Figure 6. Rows from top to bottom display (1st) the input image; (2nd) the ground truth segmentation; (3rd) the abnormality map as a KDE using our self-supervised training approach. Results are provided for five categories with three randomly selected images from the testing data.
